# B cell depletion improves therapeutic index of combination checkpoint blockade in patients with advanced melanoma

**DOI:** 10.1172/JCI205606

**Published:** 2026-08-25

**Authors:** Kavita M. Dhodapkar, Antonio Matera, Alyssa M. Duffy, Azmain Taz, Renee Julia Manalo, Melinda Yushak, Ragini Kudchadkar, David H. Lawson, Madhav V. Dhodapkar

**Affiliations:** 1 Department of Translational Science and Therapeutics, Fred Hutchinson Cancer Center, Seattle, Washington, USA.; 2 Seattle Children’s Hospital, Seattle, Washington, USA.; 3 Winship Cancer Institute, Emory University, Atlanta, Georgia, USA.

**Keywords:** Immunology, Oncology, Immunotherapy, Melanoma

## Abstract

**BACKGROUND:**

Combined checkpoint blockade (CCB) of programmed-death–1 (PD-1) and cytotoxic-T-lymphocyte–associated protein-4 (CTLA-4) is highly active in melanoma but limited by significant morbidity from immune-related adverse events (irAEs). Effective strategies to prevent CCB-mediated irAEs are lacking.

**METHODS:**

Patients with advanced melanoma were randomly assigned to receive standard of care ipilimumab and nivolumab alone (Arm A: ipi/nivo, *n* = 7) or with one cycle of rituximab (Arm B; ipi/nivo+rituximab, *n* = 7).

**RESULTS:**

Patients receiving ipi/nivo+rituximab experienced lower rates of greater than grade-3(G3) irAEs (14% versus 57%) and superior G3-irAE–free survival compared with those in ipi/nivo arm (2-year G3-irAE-free survival 86% versus 29%, *P* = 0.01), without adverse impact on tumor regression or survival. G3 hypersensitivity reactions to rituximab (43% in Arm B) prompted trial closure. Rituximab depleted pretherapy activated naive B cells linked to autoimmunity and enhanced CCB-mediated induction of myeloid inflammation and CXCL13^+^ICOS^+^ CD4 T cells.

**CONCLUSION:**

B cell depletion favorably modulates CCB-mediated immune activation and may reduce irAE risk.

**TRIAL REGISTRATION:**

ClinicalTrials.gov NCT03719131.

**FUNDING:**

NIH CA238471, AR077926, CA289978, and CA290302.

## Abstract 2

Depletion of B cells led to reduced risk of immune side effects and improved immune activation following combination checkpoint therapy in melanoma patients.

## Graphical abstract

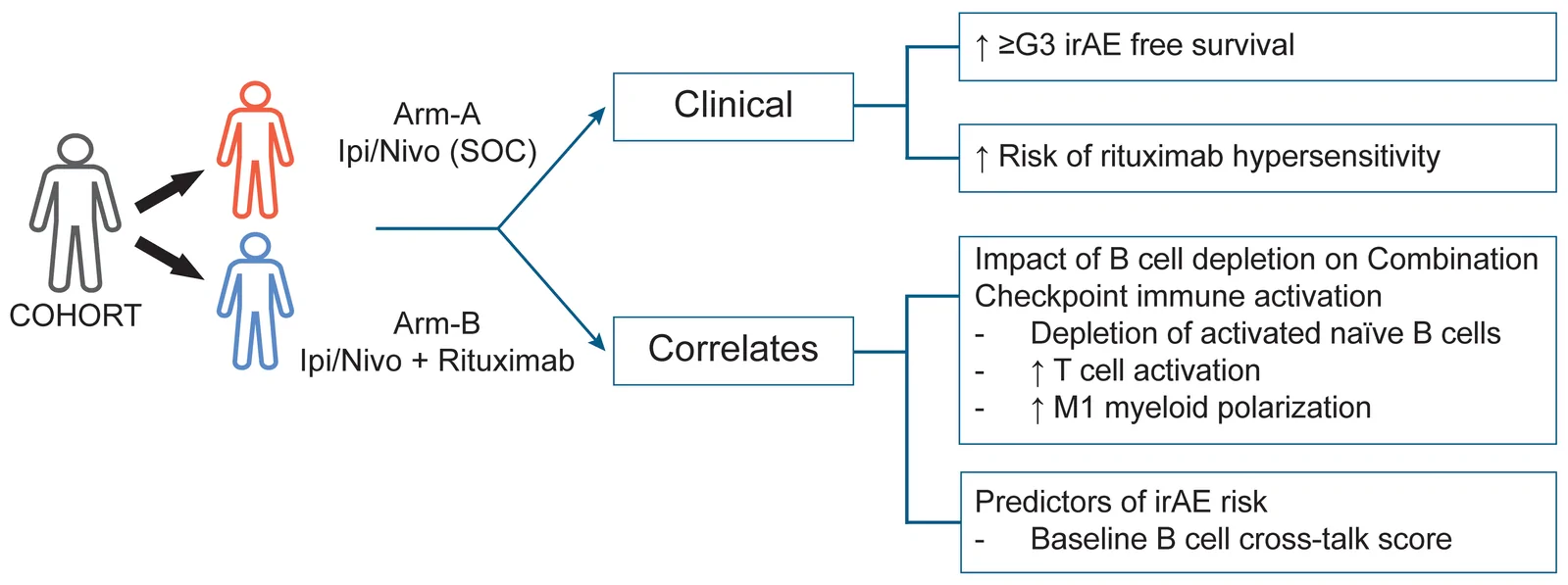

## Introduction

Combination checkpoint blockade (CCB) of CTLA-4 and PD-1 checkpoints leads to durable tumor regressions and is approved for therapy of melanoma and several other cancers (1). However, patients who are CCB treated commonly experience immune-related adverse events (irAEs), resulting in high rates of treatment discontinuation (2, 3). Current management of CCB-related toxicities is largely reactive, with monitoring for the emergence of clinical irAEs and subsequent initiation of immune-suppressive therapies (3). This strategy, however, leads to significant morbidity; preemptive strategies to reduce irAE risk remain an unmet need.

CCB leads to distinct effects on immune cells in vivo compared with blockade of individual checkpoints (4, 5). Prior studies have shown that CCB-induced B cell changes correlate with and precede the emergence of irAEs by several weeks (6). Both B and T cells, as well as interactions between these cell types, are implicated in irAE pathogenesis (3, 7). B cell depletion has led to lasting remissions in clinical autoimmunity, creating an immune reset (8). B cells have been postulated to have both positive and negative effects on tumor immunity; however, the impact of preemptive B cell depletion in patients with melanoma receiving CCB has not been tested in a randomized study. Here, we describe data from a randomized trial testing the clinical/immunologic effect of B cell depletion in patients with melanoma receiving CCB therapy.

## Results

Patients with advanced melanoma were randomly assigned to receive ipilimumab and nivolumab (ipi/nivo) (Arm A), or with one cycle (4 weekly doses) of rituximab concurrent with the initiation of immune therapy (Arm B) (Figure 1A). Primary endpoint was the rate of greater than or equal to grade3 (G3) irAEs (NCI CTCv5). G3 irAEs were observed in 4 of 7 patients in Arm A and 1 of 7 patients in Arm B. G3-irAE-free survival at 2 years was significantly higher in Arm B (Arm A 29% versus Arm B 86%, *P* = 0.01; Figure 1B). Reduction in G3-irAE was not based on any specific organ/tissue (Figure 1C and Supplemental Table 1; supplemental material available online with this article; https://doi.org/10.1172/JCI205606DS1). Rate of objective tumor response (>PR) was similar in both arms Arm A 29%, (95% confidence interval (CI) 5%–64%) versus Arm B 43%, (95% CI 16%–75%) (Table 1). Patients in Arm B experienced modest increase in progression-free survival (Figure 1D) and overall survival was similar in both arms (Figure 1E). Patients in Arm B, however, experienced transient and fully reversible rituximab-mediated hypersensitivity, with G3 reactions in 3 of 7 (43%) patients (Table 1). Hypersensitivity to rituximab occurred with the first dose in 4 patients and with subsequent doses in 2 additional patients. The study was prematurely closed to accrual due to a higher than expected rate of rituximab-mediated hypersensitivity observed in Arm B.

Paired pre- and posttreatment blood samples obtained at 4 weeks following start of CCB therapy were analyzed by mass cytometry and single-cell transcriptomics as orthogonal methods to evaluate the impact of B cell depletion on CCB-mediated immune effects. As expected, rituximab led to B cell depletion, particularly involving naive B cells with relative enrichment of CD19^+^ plasmablasts among residual B cells in posttreatment samples (Figure 1, F–H). In serial specimens, B cell depletion following a single cycle of rituximab was transient, as expected, and returned to baseline by 12 months (Supplemental Figure 1). Together, these data show that rituximab-mediated B cell depletion in patients undergoing CCB leads to improved therapeutic index with reduced risk of CCB-induced G3-irAEs.

B cell depletion was the most prominent compositional change between the treatment arms, as analyzed by single-cell transcriptomics, corroborating mass cytometry data (Figure 2A). We therefore hypothesized that B cell depletion would disrupt preexisting cell-cell interactions with B cells and utilized cellchat analysis (9) to study potential cell-cell interactions with B cells. Interactions with pretherapy B cells as senders were markedly different in patients in the control arm who developed G3-irAEs compared with those who did not (Figure 2B and Supplemental Figure 2). Further analysis of top interaction modules identified 6 gene interactions (CXCL16, ICOSLG, NAMPT, CLEC2B, CD69, CD55) whose expression on B cells was linked to G3-irAEs (Figure 2C). Integration of these genes into a B cell crosstalk score correlated with higher irAE risk in pretreatment B cells in Arm A (Figure 2D). Expression of this signature was enriched in a distinct subset of activated naive B cells (Figure 2E and Supplemental Figure 3), with similarity to activated naive B cells previously implicated in autoimmunity (10). Rituximab therapy led to reduced irAE risk in spite of similar baseline B cell cross-talk scores in both arms (Supplemental Figure 4). These data suggest that B cell depletion may exert its effects in part by disrupting preexisting B cell alterations and T:B cell cross-talk. Analysis of an independent dataset (11) further confirmed a correlation between baseline B cell cross-talk score and irAE risk in patients with melanoma receiving CCB (Supplemental Figure 5).

We next focused on how B cell depletion impacted CCB-induced immune changes, focusing on T and myeloid cells. There were no significant compositional differences in major immune cell types besides B cells (Supplemental Figure 6). FlowSOM analysis of CD4^+^ and CD8^+^ T cells by mass cytometry identified 10 metaclusters (MC) (Figure 3, A and B, and Supplemental Figure 7). CCB led to expansion of MC8; proliferating memory CD4^+^ T cells with a HLADR^hi^CD38^+^ICOS^+^FOXP3^–^ phenotype (Figure 3A), particularly in Arm B. Among CD8^+^ T cells, MC9 (DR^+^CD27^+^CD28^+^GranzymeB^+^) proliferating memory CD8^+^ T cells were increased following CCB in both arms (Figure 3B). Transcriptomic analyses identified 25 clusters of CD4^+^/CD8^+^ T cells (Figure 3C, Supplemental Figure 8A, and Supplemental Table 2). Of these, a distinct cluster of CD4^+^ T cells (CXCL13^+^ICOS^+^) (Figure 3D and Supplemental Figure 8B) and effector CD8^+^ T cells expressing cytolytic markers (Figure 3D and Supplemental Figure 8C) were significantly increased in Arm B. Together, these data show that B cell depletion leads to increase in CCB-mediated induction of distinct T cell subsets, particularly CXCL13^+^CD4^+^ T cells.

Hallmark analysis of CCB-induced differentially expressed genes (DEGs) in monocytes revealed that CCB therapy in the setting of B cell depletion in Arm B was associated with enhanced inflammatory signaling (Figure 3, E and F, and Supplemental Table 3). This included several genes in the activated NF-κB pathway, leading to increased NF-κB module signature (Biocarta) (Figure 3G and Supplemental Figure 9, A–C). These changes were observed across multiple monocyte subsets (Supplemental Figure 9, A–C). As enhanced NF-κB activation is a feature of canonical M1 macrophage polarization, we compared these changes with published M1 signatures (12). Posttreatment myeloid cells in Arm B exhibited a higher M1 signature compared with those in Arm A. These data show that CCB in the setting of B cell depletion leads to greater myeloid inflammation and M1 polarization (Supplemental Figure 9D and Supplemental Table 4).

## Discussion

These data demonstrate that B cell depletion leads to enhanced CCB-mediated T and myeloid activation and may lead to clinically meaningful reduction in irAE risk. To our knowledge, these are the first prospective randomized clinical studies evaluating the clinical and immunologic impact of B cell depletion on patients with melanoma receiving CCB therapy. The mechanism underlying reduced irAE risk observed herein may relate to rituximab-mediated depletion of activated naive B cells previously linked to autoimmunity (10). Preemptive B cell depletion surprisingly also enhanced CCB-mediated induction of CXCL13^+^/ICOS^+^CD4^+^ T cells, previously implicated in tumor immunity (13) and implicated in the efficacy of CTLA4 blockade (14), a critical component of this combination. The impact of preemptive B cell depletion on CCB-induced myeloid inflammation is also of interest, with emerging data about the contribution of myeloid cells in CCB effects (15). An important strength of these data is the prospective randomized design. An important limitation is the small numbers of patients treated, which limits the power of this preliminary observation. While the 2 cohorts were generally comparable, irAE reduction in the rituximab arm was observed in spite of more females in Arm B, previously linked to higher irAE risk. Promising clinical results observed in this study implore the need for further clinical investigation of preemptive B cell depletion in CCB therapy. Increased risk of rituximab hypersensitivity with CCB initiation would also need to be addressed and could be reduced by limiting all rituximab dosing to prior to CCB initiation or utilizing alternate B cell depletion strategies. Increased hypersensitivity reactions observed in this trial however support potential role of CTLA4/PD1 checkpoints in human allergy and investigation of CTLA4 agonists in allergic diseases (16). B cell depletion has led to durable responses in several human autoimmune diseases by inducing an immune reset (8). The apparent durability of irAE-risk reduction observed in this study is reminiscent of a similar immune reset phenomenon that deserves further investigation. Preemptive B cell depletion may not only reduce irAE risk and improve CCB-mediated immunity but also allow patients with cancer with prior autoimmunity to benefit from CCB therapy (17).

## Methods

### Sex as a variable.

The study enrolled both male and female patients.

### Clinical trial.

Patients with unresectable advanced melanoma were enrolled in a prospective randomized clinical trial to test the efficacy of preemptive rituximab in reducing immune-related adverse events (irAEs) in patients with melanoma receiving combination checkpoint blockade as standard of care (clinical trial protocol in appendix). The trial was registered in clinicaltrials.gov under NCT03719131. Key eligibility criteria included patients with a diagnosis of unresectable advanced melanoma clinically felt suitable to receive combination checkpoint blockade. Patients were required to have adequate hematologic (white blood count > 3,000/μL, absolute neutrophil count >1,500/μL, platelet count > 100,000/μL, hemoglobin > 9 g/dL), renal (serum creatinine <1.5 x ULN or creatinine clearance (CrCl) > 40 ml/min) and hepatic function (transaminases <3 x upper limit normal (ULN) or <5 x ULN for patients with documented liver metastases, alkaline phosphatase < 2X ULN or <5XULN for those with bone metastasis, total bilirubin < 1.5 X ULN except those with direct bilirubin or Gilbert’s syndrome) and serum lactate dehydrogenase <10 X ULN. Patients with prior hypersensitivity to rituximab or known central nervous system metastatic disease / leptomeningeal involvement were excluded. Patients with active, known or suspected autoimmune disorders including lupus and type I diabetes, active disease or history of inflammatory bowel disease, or non-replacement steroid use were ineligible. Clinical protocol is included in the Appendix. Primary objective was to compare rates of Grade3 or greater irAEs in the two treatment arms.

Combination checkpoint blockade was administered as per current standard of care for advanced melanoma, consisting of nivolumab at a dose of 1 mg/kg and ipilimumab at a dose of 3 mg/kg every 3 weeks for 4 doses, followed by nivolumab maintenance at a dose of 480 mg every 4 weeks for 1 year. Patients were randomly assigned to no additional therapy (Arm A), or 4 weekly doses of rituximab for B cell depletion (Arm B). Rituximab was administered as 375 mg/m^2^ intravenously for the first dose followed by weekly subcutaneous dosing at 1400 mg dose of Rituxan Hycela for weeks 2-4, as per dosing utilized in lymphoma. All patients were clinically monitored for the development of irAEs utilizing NCI Common Toxicity Criteria v5.0. All patients who received at least one dose of study drug were included in safety analysis, and safety assessment continued through treatment phase and up to 100 days after the last dose of study drug. Tumor response was assessed according to the Response Evaluation Criteria in Solid Tumors (RECIST v1.1) at 12 weeks and every 12 weeks thereafter. The study was initially designed to enroll 22 patients per treatment arm. After initial observations of rituximab-hypersensitivity reactions, the protocol was first amended to delay initiation of rituximab to a week after initiation of CCB therapy. However, due to continued observation of Rituxan hypersensitivity, the study was closed to accrual after enrollment of 15 patients. The study was open between June 2019 and April 2025. The current report includes all patients treated in the trial.

### Ethical approval and informed consent.

The study was conducted in accordance with the Declaration of Helsinki and approved by Emory University Institutional Review Board. Written informed consent was obtained from all patients prior to participation.

### Sample collection.

Peripheral blood (PB) samples were collected at predefined time points including before therapy (baseline) and at 4 weeks after initiation of therapy. Peripheral blood mononuclear cells (PBMCs) were isolated by density gradient centrifugation using Ficoll-Paque PLUS (GE Healthcare), following standard protocols. PBMCs were cryopreserved and stored in liquid N2. Cryopreserved PBMCs were thawed prior to analyses. 14 patients were enrolled in the study. Specimens from 12 patients were analyzed by mass cytometry and by single cell multiomics in 11 patients (Supplemental Table 5).

### Immunophenotyping with single-cell mass cytometry (CyTOF).

Paired pre- and posttreatment blood specimens were analyzed by mass cytometry to evaluate changes in immune cells during therapy. Immunophenotyping was performed utilizing a 41-marker mass cytometry panel (Supplemental Table 6). PBMCs were stained with cell surface antibodies, fixed, permeabilized, and washed in accordance with the manufacturer’s cell-surface and nuclear staining protocol as previously described (18). After antibody staining, cells were incubated with intercalation solution, mixed with EQ Four Element Calibration Beads and acquired using a Helios mass cytometer (all from Standard Biotools). Gating and data analysis were performed using Cytobank (https://www.cytobank.org). Dead cells and doublets were excluded using cisplatin intercalator and DNA content with iridium intercalator.

### Single cell transcriptomics.

Pre/post treatment PBMCs were thawed and incubated at 37°C for 1 hour to rest. Cells were stained using a TotalSeq-C antibody cocktail following the 10X Genomics protocol for Chromium Single-Cell Immune Profiling with Feature Barcoding Technology (ver. 1.0). Single cells were isolated using the Chromium Controller (10X Genomics). Gene expression and CITE-seq libraries were prepared using the following kits from 10× Genomics: Chromium Next GEM Single Cell 5’ Kit v2 (PN-1000263), Chromium 5’ Feature Barcode Kit (PN-1000541), Chromium Single-Cell 5′ Library Construction Kit (PN-1000190), Chromium Next GEM Chip K Single Cell Kit (PN-1000287), Dual Index Kit TT Set A (PN-1000215) and Dual Index Kit TN Set A (PN-1000250). Sequencing was conducted on an Illumina NovaSeq 6000 at a targeted depth of 50,000 reads/cell for GEX libraries and 5000 reads/cell for CITE-seq libraries.

### Data preprocessing.

Raw sequencing data were processed with Cell Ranger Multi (10x Genomics, v7.1.0) to jointly align reads, perform barcode and UMI counting, and generate filtered feature–barcode matrices for both RNA and ADT modalities. The GRCh38 human reference genome and the corresponding feature barcode reference were used for alignment. Filtered count matrices were then used as input for downstream analyses in R.

### Single-cell data analysis.

All downstream analyses were performed in R (v4.4) using Seurat (v5.1.0). Doublets were identified with DoubletFinder (v2.0.6), and only singlets were retained. After doublet removal, cells were filtered by quality-control thresholds: nFeature_RNA > 200, nFeature_RNA < 3,000, and percent.mt < 10%. RNA and ADT counts were normalized, and the stabilized variance of each gene was computed using the variance-stabilizing transformation (VST) to support principal component analysis (PCA).Sample integration and batch correction were performed using Canonical Correlation Analysis (CCA) to define a shared low-dimensional space. The first 50 principal components were used to capture the main axes of transcriptional variability. Dimensionality reduction for visualization was performed with Uniform Manifold Approximation and Projection (UMAP), and unsupervised community detection was achieved using the Louvain algorithm, as implemented in Seurat. A shared nearest neighbor (SNN) graph was constructed from the PCA space to identify distinct cellular clusters. For multimodal integration of RNA and ADT modalities, Weighted Nearest Neighbor (WNN) analysis was applied to jointly model transcriptomic and proteomic variation in a unified latent space. Cell identities were first assigned by reference-based label transfer (*MapQuery* function in Seurat) using the pbmc_multimodal_2023 dataset as reference. Differential expression analyses were performed for both RNA and ADT modalities to identify cluster-specific markers and surface proteins distinguishing major immune populations and experimental conditions. Cluster annotations were then manually refined based on canonical RNA and surface protein markers. Statistical testing used non-parametric Wilcoxon rank-sum or Mann–Whitney U tests with false discovery rate (FDR) correction.

### Lineage-specific analyses.

To obtain purified cellular subpopulations, lineage-specific selection strategies were applied. B cell and myeloid lineages were isolated using annotation labels from the pbmc_multimodal_2023 reference, selecting the following subsets: *B intermediate*, *B memory*, *B naive*, *Plasmablast*, and *ASDC*, *CD14 Mono*, *CD16 Mono*, *cDC1*, *cDC2*, *pDC*, respectively. T cells were isolated based on ADT expression of CD3 > 1.1, whereas NK cells were identified using CellSelector function from Seurat on the UMAP to select NK/T clusters, followed by gating for CD3 < 1. Expression of canonical surface protein markers was visualized using UMAP feature plots to support cell type annotation (Supplemental Figure 10). Each lineage was reprocessed through CCA-based integration and downstream multimodal analyses for clustering and marker identification, as detailed above.

### Cell-cell interaction analysis.

Cell-cell communication was inferred using CellChat (9). Analyses were performed on a subset of the dataset restricted to pre-treatment samples from treatment Arm A, comparing patients with G3 (>grade3) immune-related adverse events (irAEs) and no G3 irAE. The analysis focused on B cells, monocytes, CD4 T cells, and CD8 T cells, which were included as interacting cell populations. B cells were specified as signal senders and cell–cell interaction probabilities were computed using default CellChat settings.

### Module scoring and pathway enrichment analysis.

Gene module activity scores were calculated using AddModuleScore_UCell function. Hallmark pathway enrichment analysis was performed using Single Cell Pathway Analysis (SCPA) (19) applying a significance threshold of log2 fold change ≥ 0.5 and p-value < 0.05. For volcano plots and heatmap visualizations, differential expression results were filtered using log2 fold change ≥ 0.2 and p-value < 0.05, excluding non-coding genes from the analysis.

### Statistics.

Wilcoxon signed-rank tests (2-sided) or Mann–Whitney *U* tests were used for paired within-patient (pre- versus posttreatment) and arm-stratified comparisons, respectively.

Benjamini–Hochberg FDR correction was applied to control for multiple testing. This analytical framework was used both globally and within each lineage-specific analysis (B, T, NK, myeloid) to assess differences in cell abundance and gene/protein expression. Visualization of cell clusters, marker expression, and all statistical results was performed using Seurat and ggplot2 in R. Statistical analysis of mass cytometry was performed using Cytobank and GraphPad Prism. Two-tailed Mann-Whitney was used to compare data between groups.

### Data availability.

The sequencing data included in this article are available on NCBI’s Gene-Expression Omnibus under accession number GSE335494. Other data are available upon reasonable request from the corresponding authors.

## Manuscript information

### Author contributions.

KMD and MVD designed and provided oversight for the entire study and wrote the manuscript. AM performed bioinformatic analyses. AMD, AT, and RJM performed correlative studies. MY, RK, and DHL performed clinical research. All authors reviewed and approved the manuscript.

### Conflict of interest.

The authors have declared that no conflict of interest exists.

### Funding.

This work is the result of NIH funding, in whole or in part, and is subject to the NIH Public Access Policy. Through acceptance of this federal funding, the NIH has been given a right to make the work publicly available in PubMed Central.

- NIH CA238471 (to KMD).
- NIH AR077926 (to KMD).
- NIH CA289978 (to MVD).
- NIH CA290302 (to MVD).
- P30CA138292 (to Winship immune monitoring resource).

## Supplementary Material

Supplemental data

ICMJE disclosure forms

Supplemental table 2

Supplemental table 3

Supplemental table 4

Supplemental table 5

Supporting data values

## Figures and Tables

**Figure 1:**
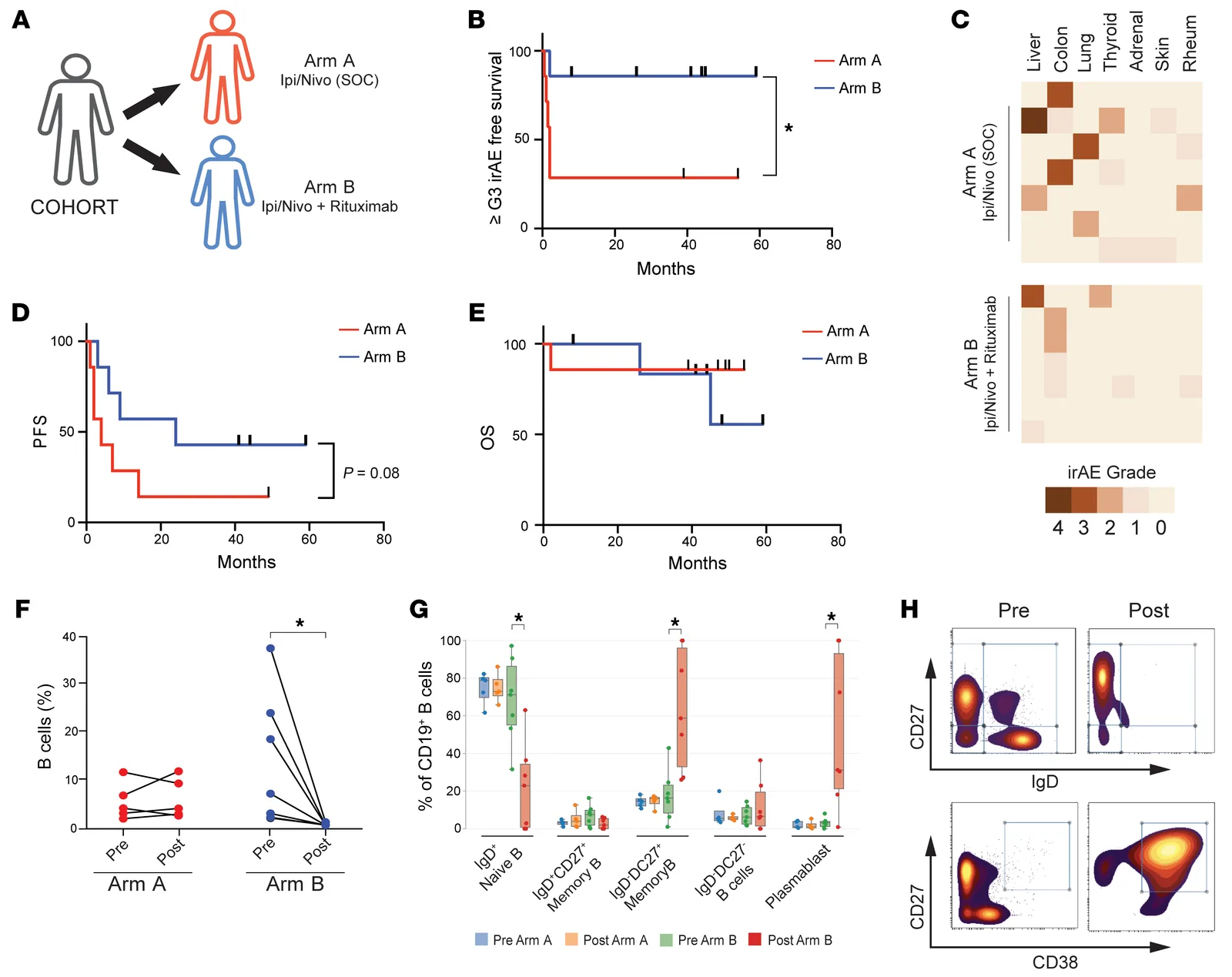
Clinical outcomes and B cell depletion following preemptive rituximab in patients with melanoma undergoing CCB. Patients were randomly assigned to receive standard of care ipilimumab (3 mg/kg)/nivolumab (1 mg/kg) (Arm A; *n* = 7) or with one cycle of rituximab (ipi/nivo/rituximab, Arm B; *n* = 7). (**A**) Study Schema. (**B**) ≥G3-irAE-free survival by treatment arm. (**C**) Heatmap showing patterns of irAE. (**D**) Progression-free survival (PFS) by treatment arm. (**E**) Overall survival (OS) by treatment arm. (**F**–**H**) Blood samples were obtained prior to starting therapy (Pre Rx Arm A and Pre Rx Arm B) or 4 weeks after first cycle of ipilimumab and nivolumab (Post Rx Arm A) or ipilimumab, nivolumab and one course of rituximab (Post Rx Arm B). Single cell mass cytometry was performed to analyze changes in B cells. (**F**) Figure shows B cell proportions as percent lymphocytes. (**G**) Changes in B cell subsets pre and 4 weeks post therapy. (**H**) Phenotype of residual CD19^+^ B cells following B cell depletion (representative patient ARM B). Dots represent individual patients. Box plots show mean ± SEM. *P < 0.05. Two-tailed Wilcoxon signed-rank test was used for statistical analysis in **F** and **G**.

**Figure 2:**
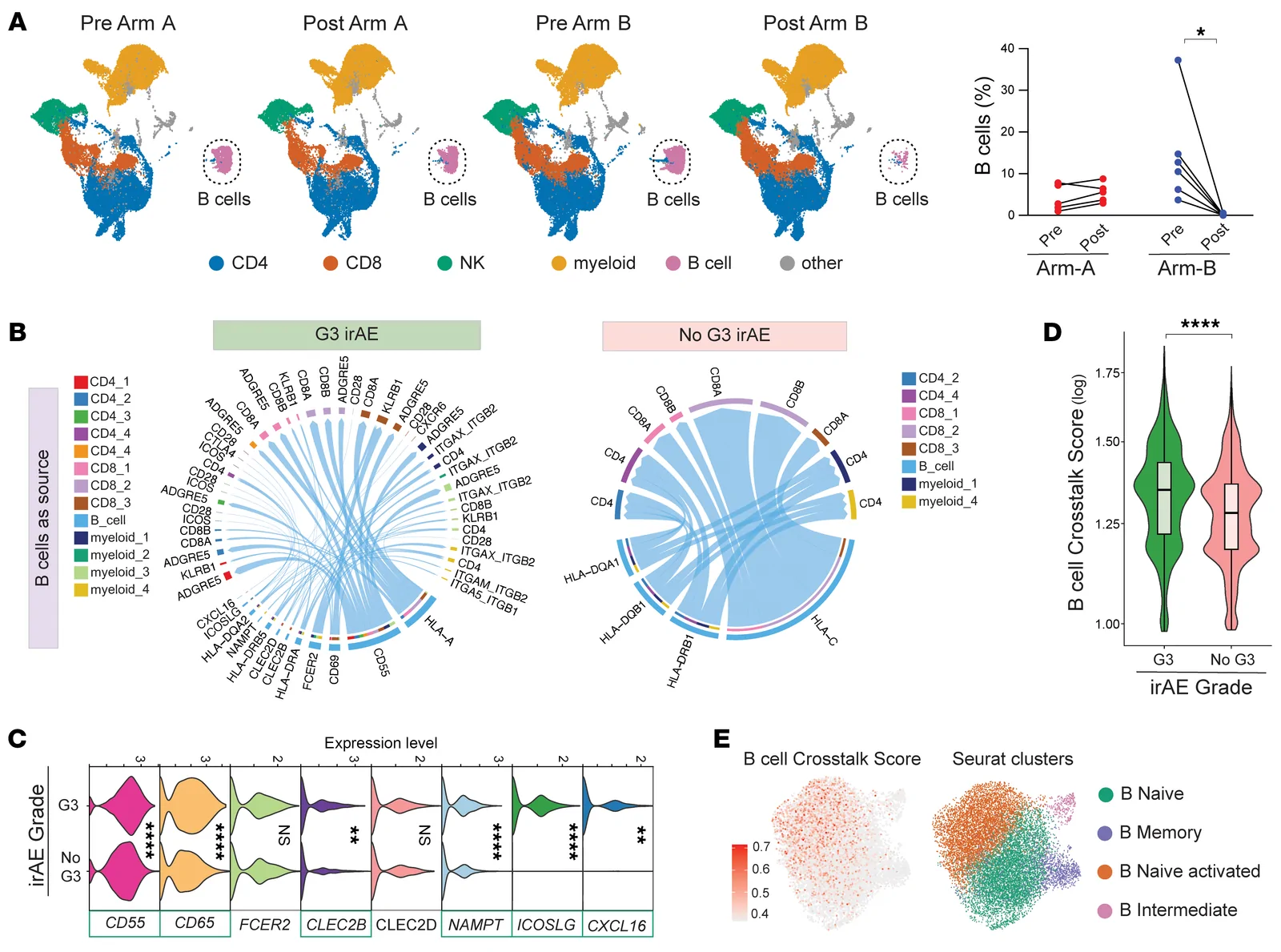
Changes in B cells and pretherapy B cell cross-talk following preemptive rituximab therapy in patients with melanoma undergoing CCB therapy. Blood samples were obtained prior to starting therapy and 4 weeks following start of cycle 1 of either CCB alone (Arm A) or with one course of Rituxan (Arm B). CITE-seq/single-cell transcriptomics was performed to analyze B cell changes and cellular interactions. (**A**) Single-cell transcriptomic analysis showing B cell depletion following rituximab therapy in Arm B, visualized by UMAP embeddings (left) and corresponding B cell proportions (right). (**B**) Chord diagram depicting B cell–centered cellular crosstalk in Arm A at pretreatment, comparing patients who developed Grade3-irAEs versus no G3-irAEs. (**C**) Expression level of differentially engaged genes on pretherapy B cells from G3-irAE and no G3-irAE irAE cohorts. (**D**) B cell crosstalk scores in Arm A stratified by irAE severity at pretreatment. (**E**) Left panel: feature plot highlights B cells with crosstalk scores in the 80th percentile (in red) and corresponding B cell clusters visualized on UMAP embeddings (right panel). Dots in A (right) represent individual patients. Box plots within the violins represent median and interquartile range in **D**. *P < 0.05.

**Figure 3:**
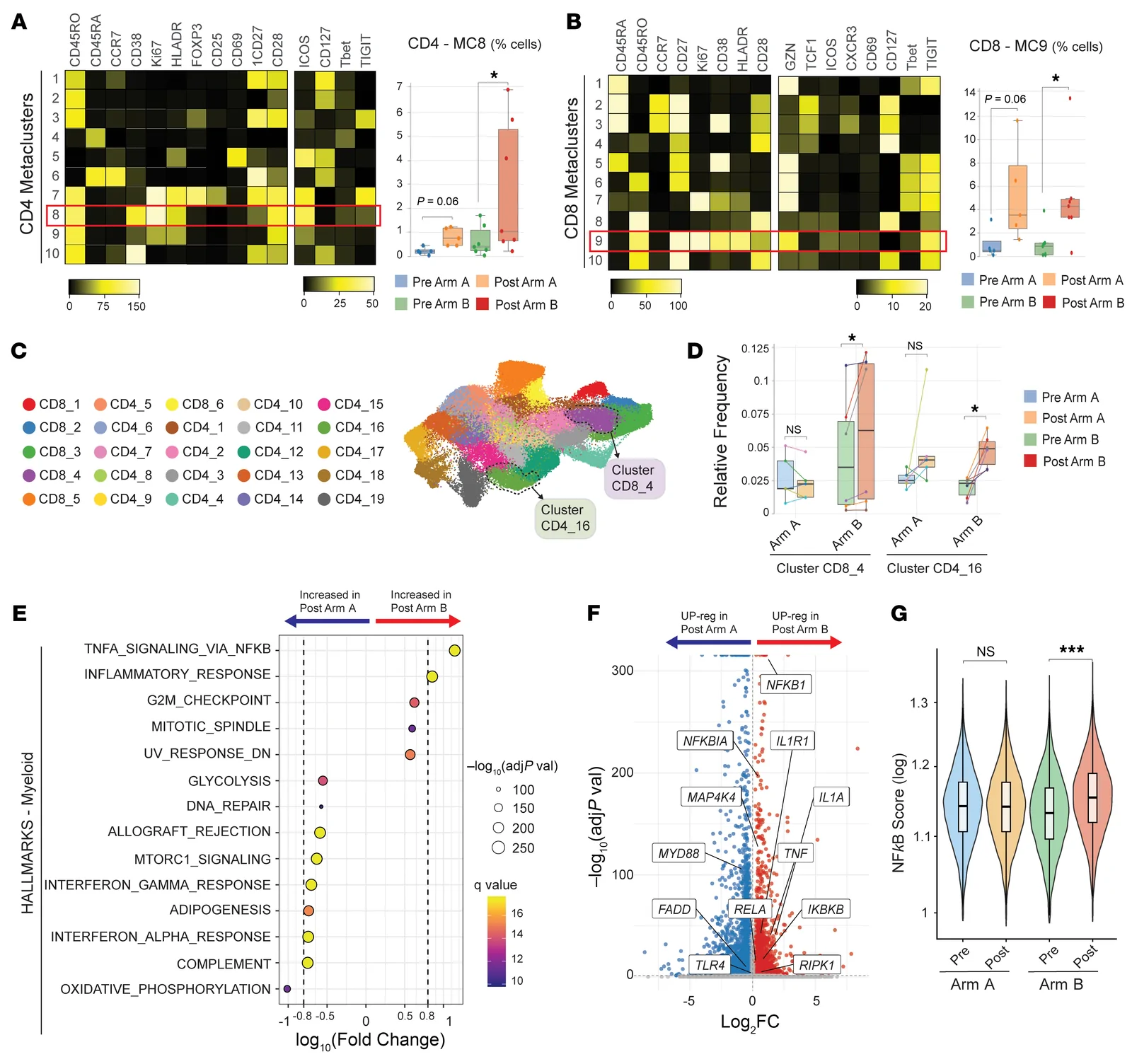
Changes in myeloid and T cell compartments following B cell depletion in patients with melanoma undergoing CCB therapy. (**A**) Heatmap (left) showing profiles of CD4^+^ T cell metaclusters analyzed by mass cytometry; Proportions of CD4 metacluster 8 increased following CCB in Arm B (right). (**B**) Heatmap (left) showing profiles of CD8^+^ T cell metaclusters analyzed by mass cytometry; Proportions of CD8 metacluster 9 increased following CCB in Arm B (right). (**C**) UMAP representation of CD4^+^ and CD8^+^ T cell clusters identified by single-cell transcriptomics. (Supplemental Table 2). (**D**) Distribution of the CD8_4 cluster and CD4_16 cluster across treatment arms. (**E**) Hallmark pathway enrichment analysis in monocytes comparing posttreatment Arm A versus Arm B. (**F**) Volcano plot showing differentially expressed genes in myeloid cells between posttreatment Arm A and Arm B; genes associated with the NF-κB signaling pathway are highlighted. (Supplemental Table 3). (**G**) NF-κB score in monocytes across treatment arms.Dots in **A**, **B**, and **D** represent individual patients. Box plots show mean ± SEM. Box plots within the violins represent median and interquartile range in **D**. *P < 0.05.

**Table 1:**
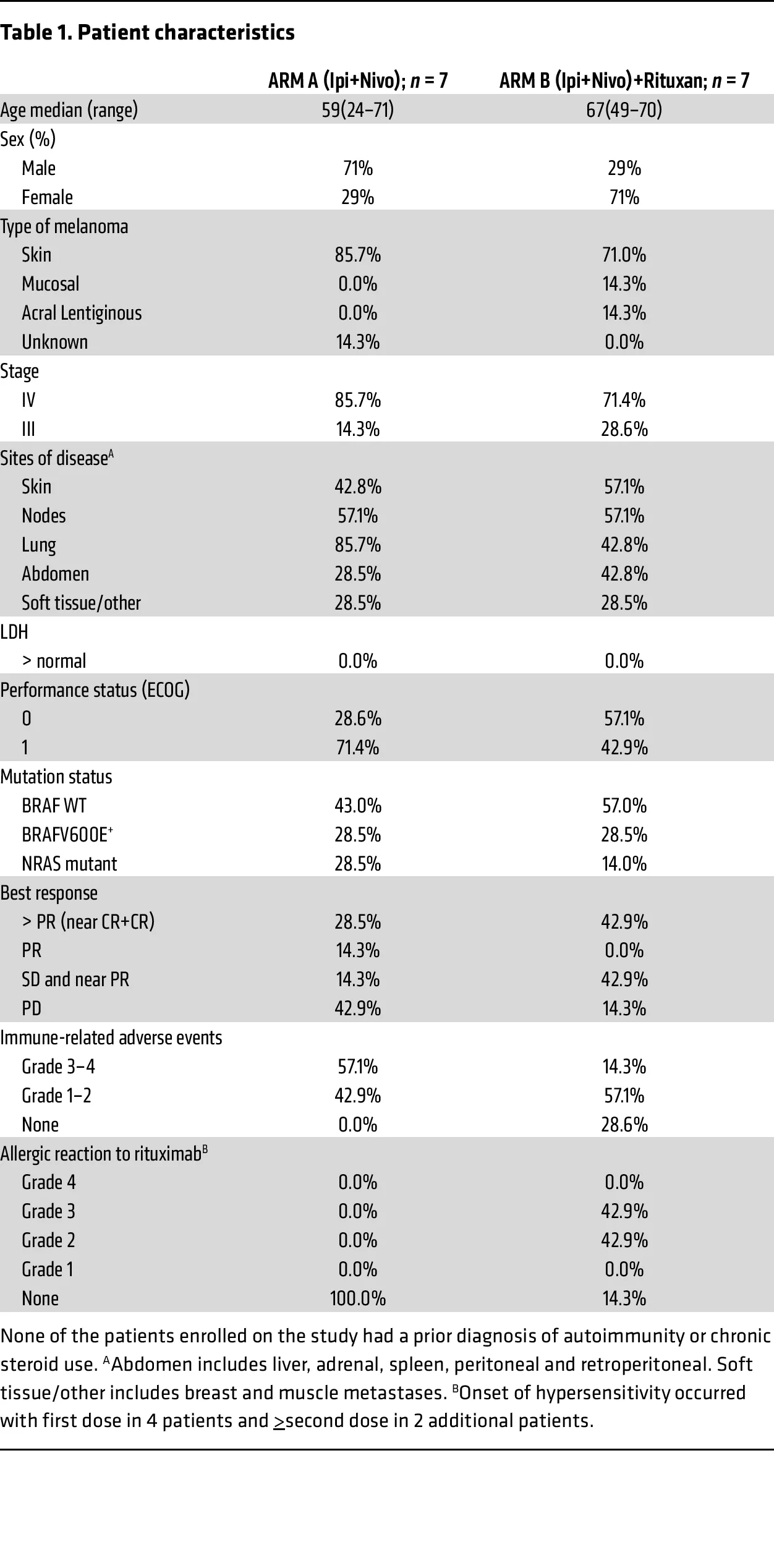
Patient characteristics

## Version 1 08/25/2026

In-Press Preview

## Version 2 11/02/2026

Electronic publication
