# Supplementary material for: B cell depletion improves therapeutic index of combination checkpoint blockade in patients with advanced melanoma
Source: J Clin Invest. 2026 Aug 25;136(21):e205606. doi: 10.1172/JCI205606 (PMC13722380; doi:10.1172/JCI205606)
Supplement: Supplementary file 1 [file jci-136-205606-s192.pdf]

Dhodapkar et al.

Supplementary Appendix:

Table of Contents

Supplementary Figures

Supplementary Figure 1. Kinetics of B cell reconstitution following rituximab treatment.
Supplementary Figure 2. Cellular clusters used for CellChat analysis
Supplementary Figure 3. Differential gene expression across B cell clusters
Supplementary Figure 4. B cell cross talk signature in treatment arms
Supplementary Figure 5. B cell cross talk signature validation in independent cohort
Supplementary Figure 6. Single-cell transcriptomic cluster frequencies
Supplementary Figure 7. Distribution of T cells in metaclusters
Supplementary Figure 8. Transcriptional and compositional changes in T cells
Supplementary Figure 9. Remodeling of myeloid cells with B cell depletion
Supplementary Figure 10. ADT expression profile

Supplementary Tables

Supplementary Table 1. Patterns of irAEs
Supplementary Table 2. Top 20 cluster identity genes for T cell clusters.
Supplementary Table 3. Differentially expressed genes in monocytes comparing post-treatment Arm-A versus Arm-B.
Supplementary Table 4. Gene set defining the M1 macrophage polarization signature.
Supplementary Table 5. Samples included in the study
Supplementary Table 6. Lists of CyTOF antibodies.

Supplementary Figure 1

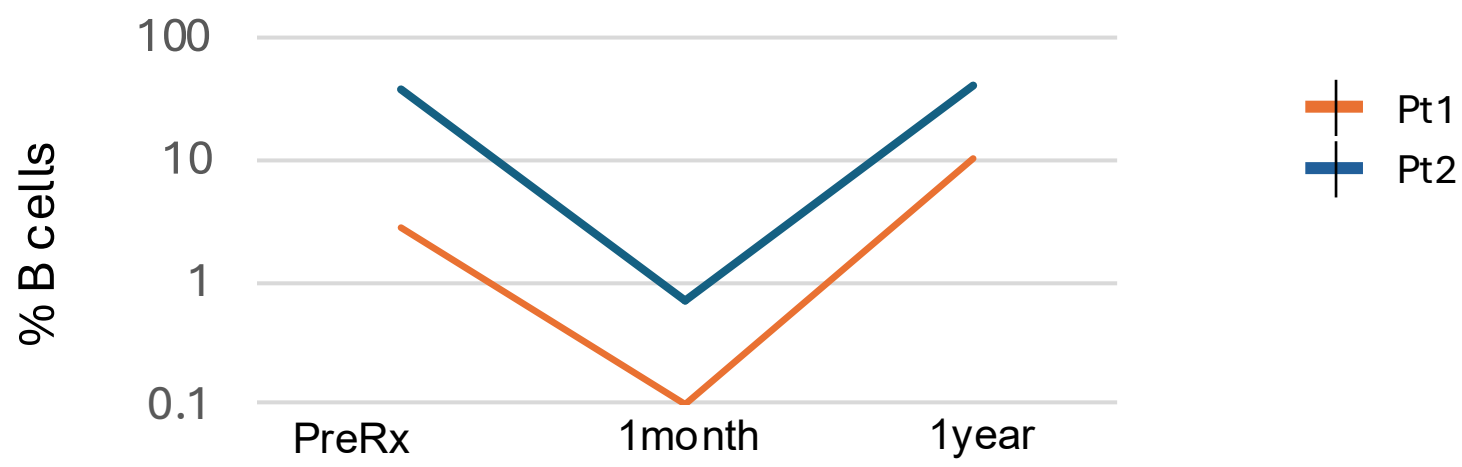

Supplementary Figure 1| Kinetics of B cell reconstitution following rituximab treatment

Representative longitudinal profiles showing B cell depletion and subsequent recovery following a single cycle of rituximab in 2 different patients on ARM-B.

Supplementary Figure 2

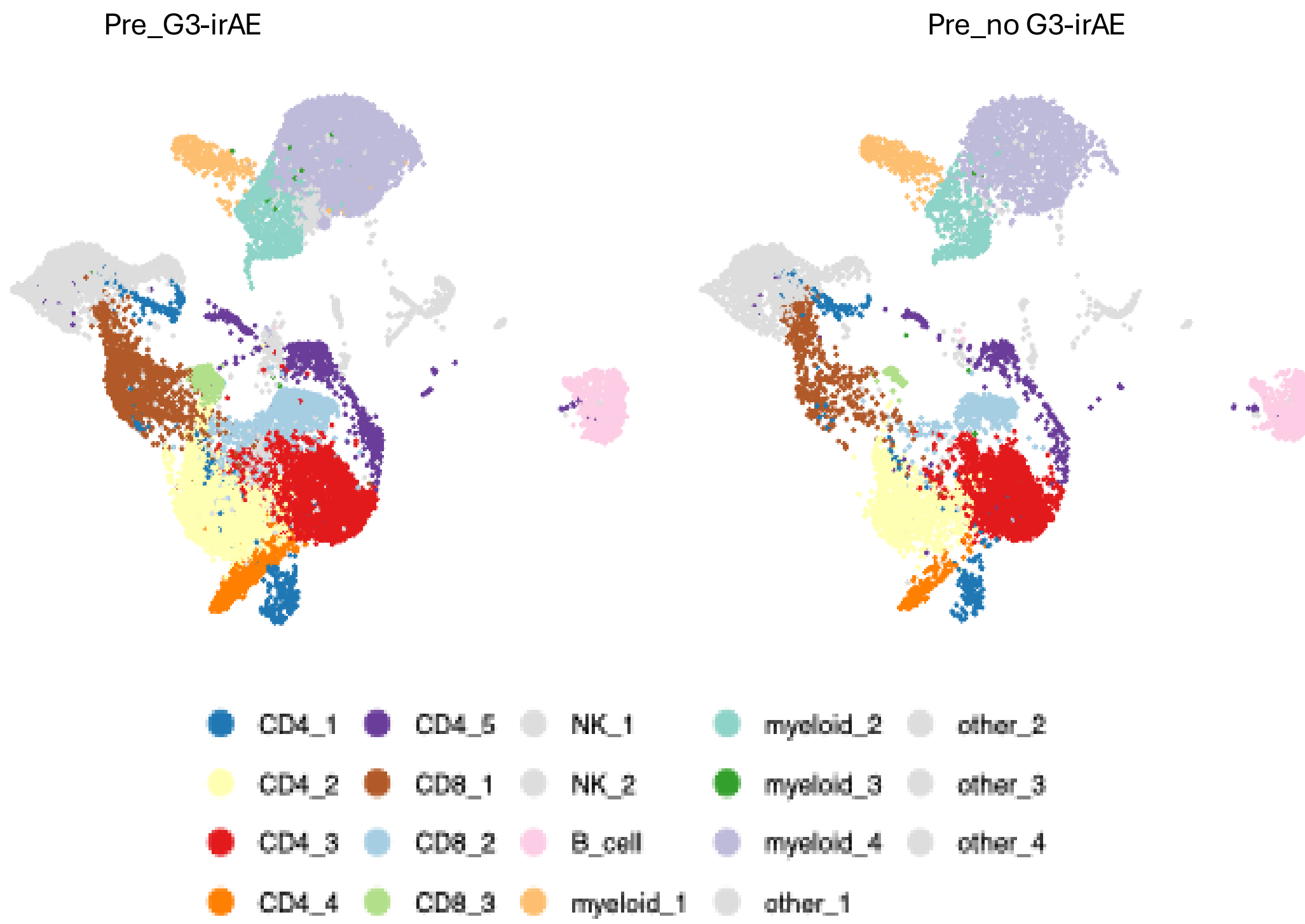

Supplementary Figure 2 | Cellular clusters and interaction networks used for CellChat analysis at pretreatment in Arm-A

UMAP representation of pretreatment Arm-A samples showing the annotated cellular clusters used as input for CellChat analysis.

Supplementary Figure 3

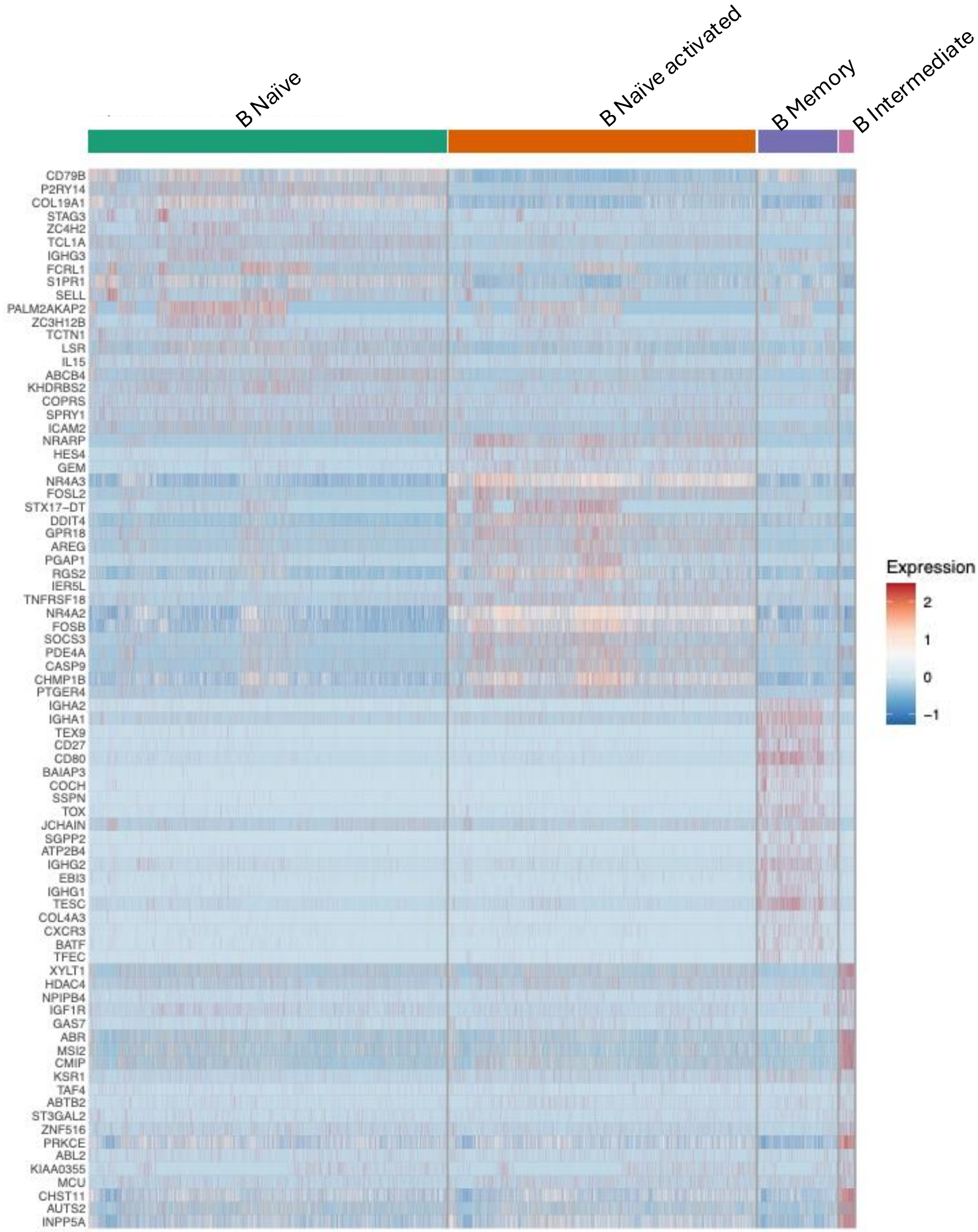

Supplementary Figure 3 | Differential gene expression across B cell clusters
Heatmap summarizing differentially expressed genes across transcriptionally defined B cell clusters.

Supplementary Figure 4

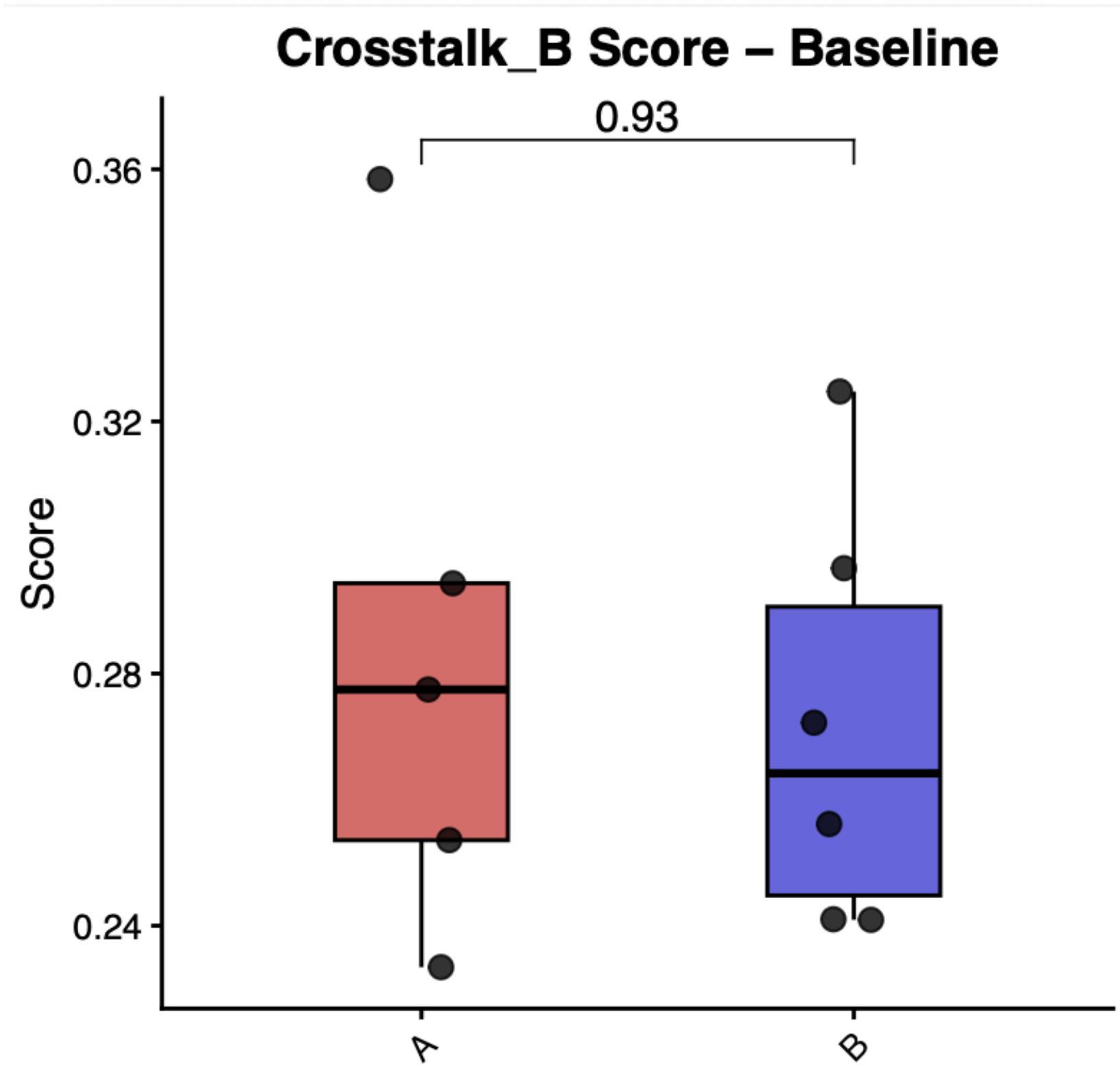

Supplementary Figure 4. Comparison of B cell crosstalk scores between treatment arms. Pseudobulk analysis of the signature_1autoB score in B cells at baseline, stratified by treatment arm A (red) and arm B (blue). Each data point represents the mean expression score calculated per individual sample. Statistical significance was evaluated using a two-sided Wilcoxon rank-sum test.

Supplementary Figure 5

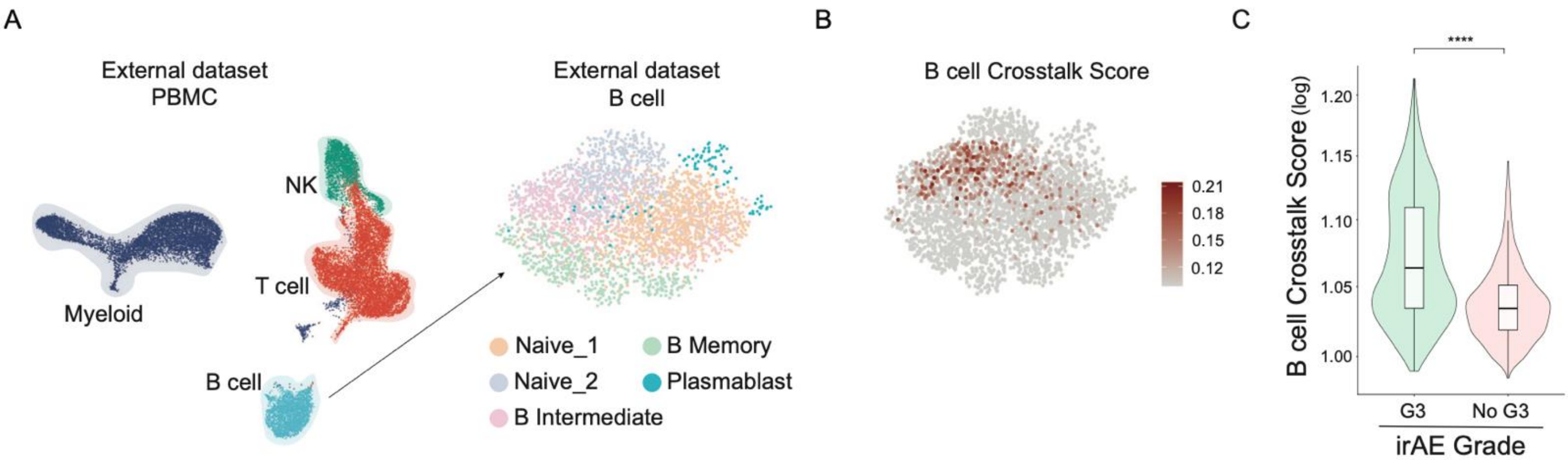

Supplementary Figure 5. B cell crosstalk signature in an independent cohort. Publicly available single-cell RNA-sequencing data from Lozano et al. (Nat Med 2022) were analyzed. PBMCs from patients who received combination checkpoint blockade therapy were integrated (n = 14; 22,462 cells).

(A) UMAP embedding of integrated PBMCs showing main immune cell compartments, including T cells, NK cells, myeloid cells, and B cells (left). UMAP embedding of re-clustered B cells (n = 2,963 cells) displaying main B cell cluster identities (right).

(B) Feature plot highlighting B cells with B cell crosstalk scores in the 80th percentile (dark red).

(**C**) Violin plot showing the distribution of the B cell crosstalk score stratified by irAE severity (G3-irAE vs. no G3-irAE).

Box plots within the violins represent the median and interquartile range in C.

****P <0.0001. Two-tailed Wilcoxon rank-sum test was used for statistical analysis in C. Statistical analyses for differential expression between main cell compartments were performed using the Wilcoxon rank-sum test with Benjamini-Hochberg correction.

Supplementary Figure 6

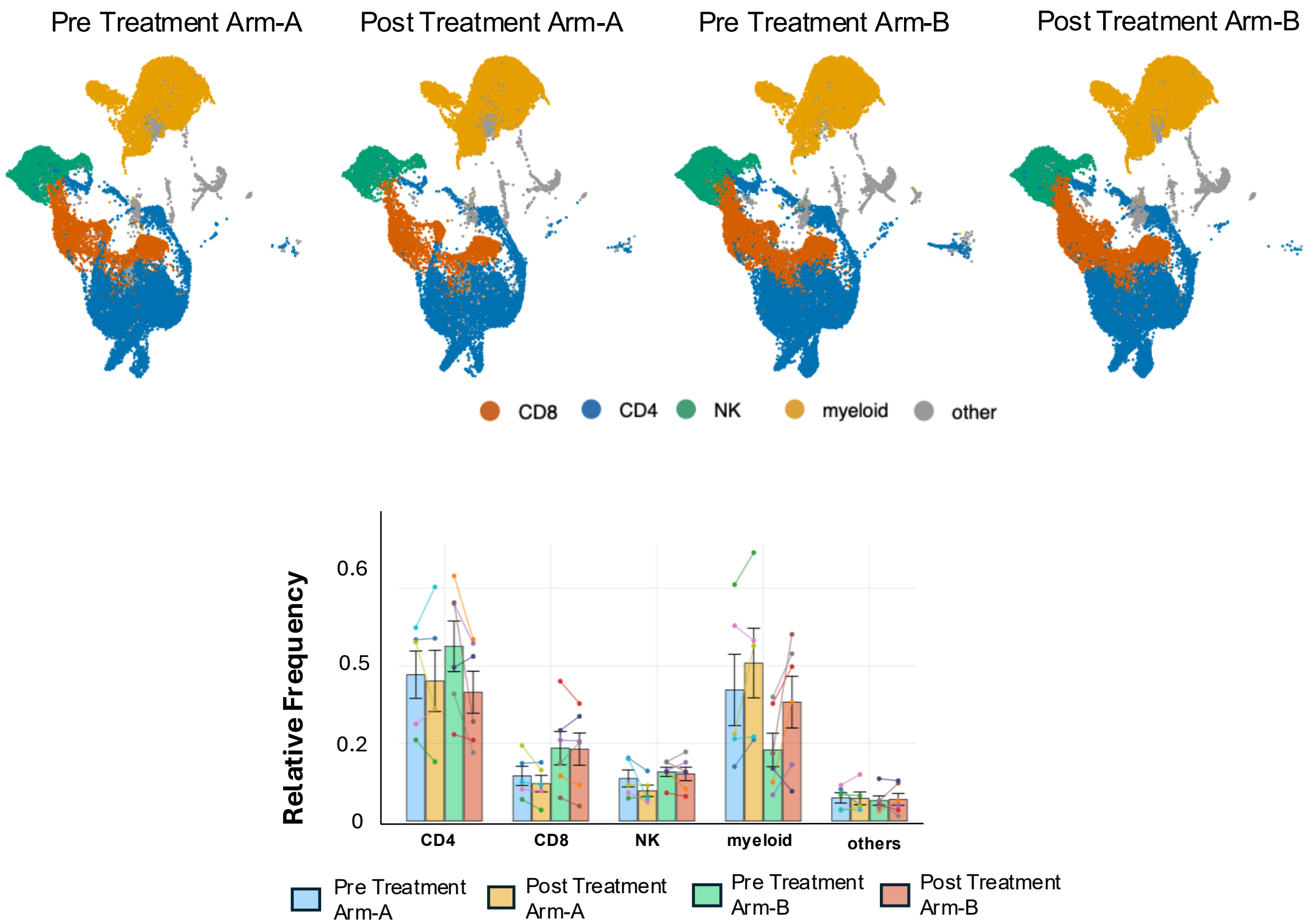

Supplementary Figure 6 | Single-cell transcriptomic cluster frequencies after exclusion of B cells

Relative frequencies of scRNA-seq–defined cellular clusters after exclusion of B cells from the analysis. UMAP embeddings (top panel) and corresponding cell proportions (bottom graph).

Supplementary Figure 7

A CD4 Metacluster proportions by arm and timepoint

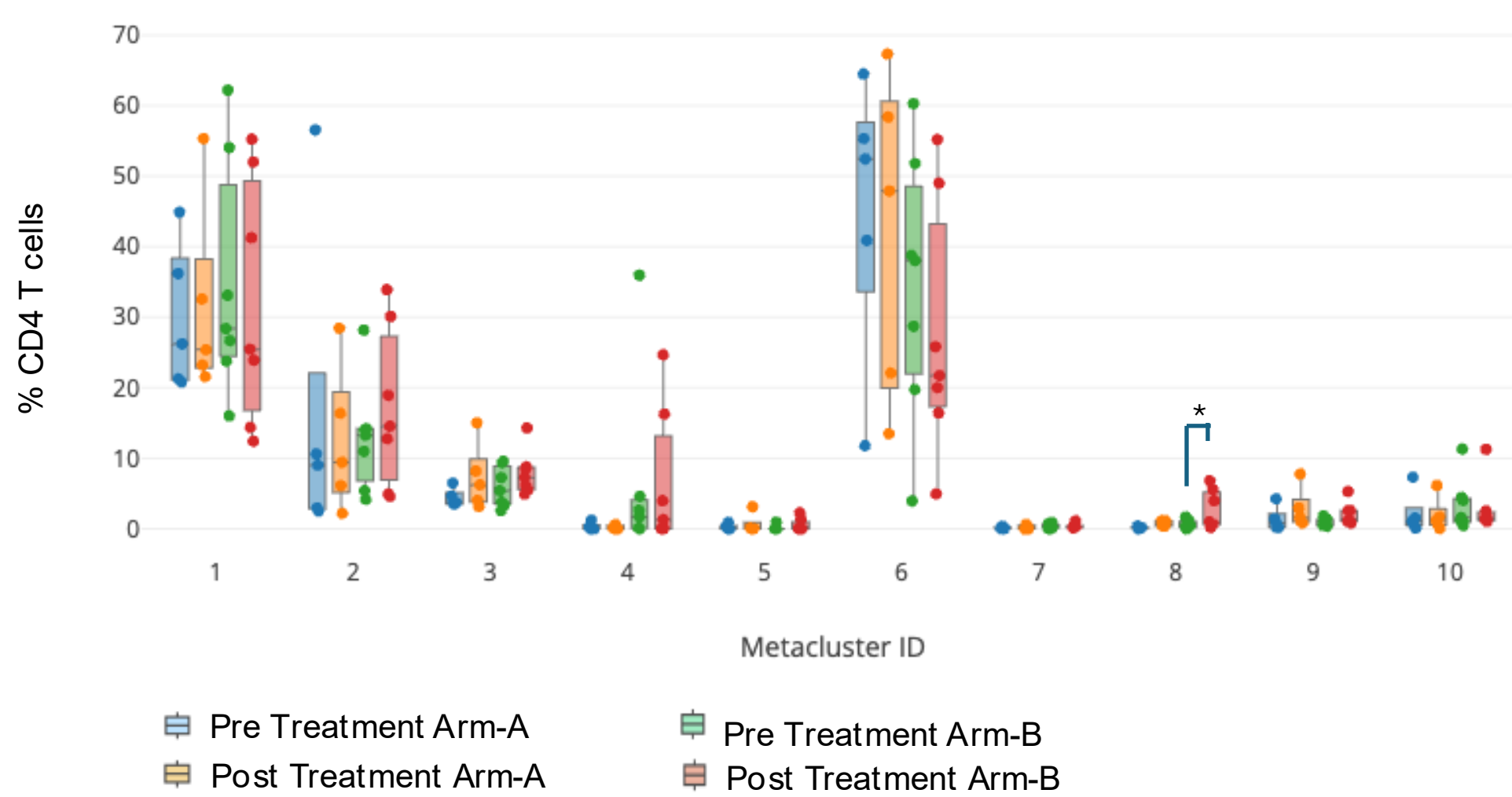

B CD8 Metacluster proportions by arm and timepoint

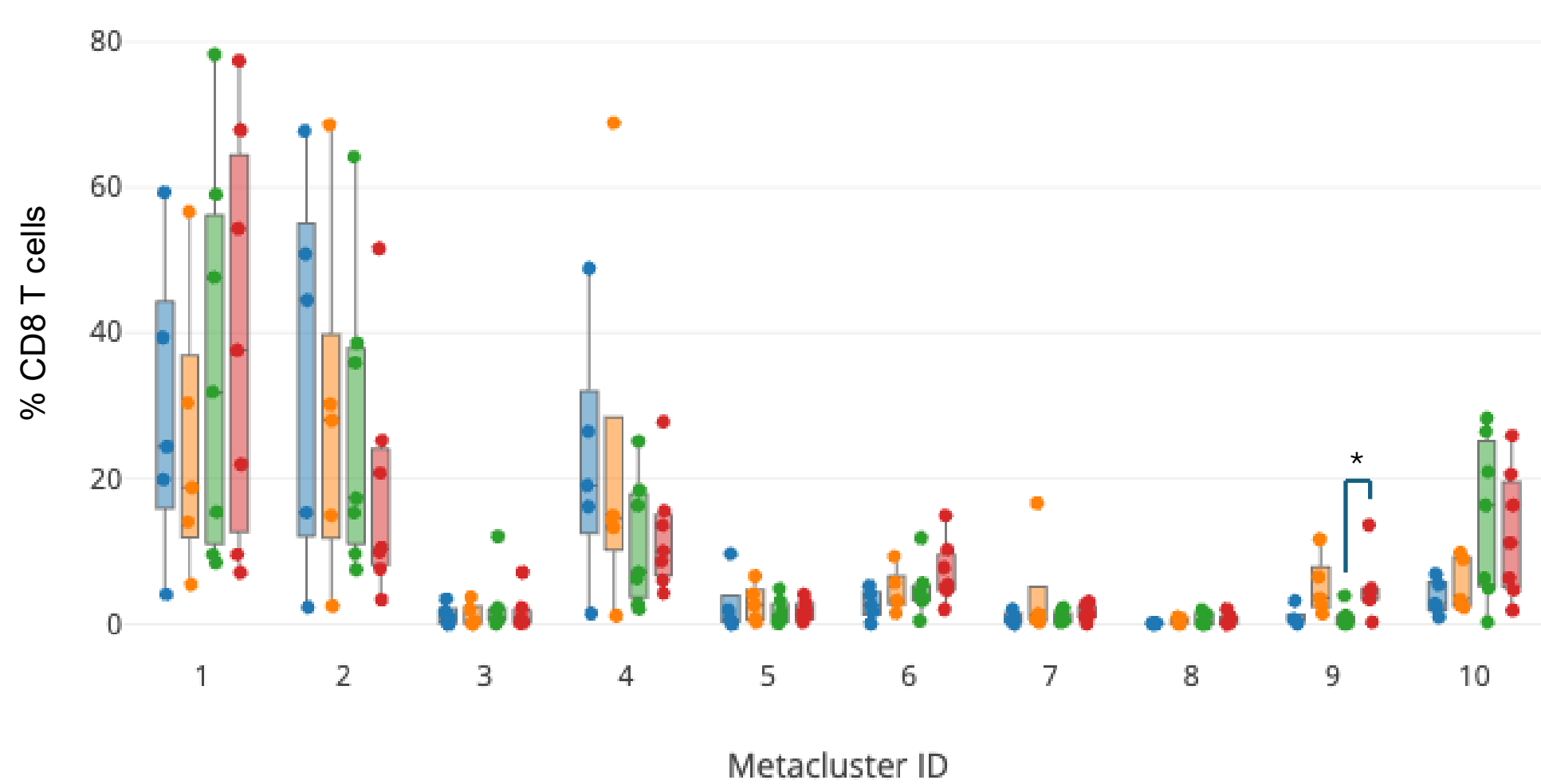

Supplementary Figure 7 | Distribution of T cells in metaclusters identified by single cell mass cytometry.

(A) CD4+ T cell proportions across 10 CD4 metaclusters.

(B) CD8+ T cell proportions across 10 CD8 metaclusters.

Supplementary Figure 8

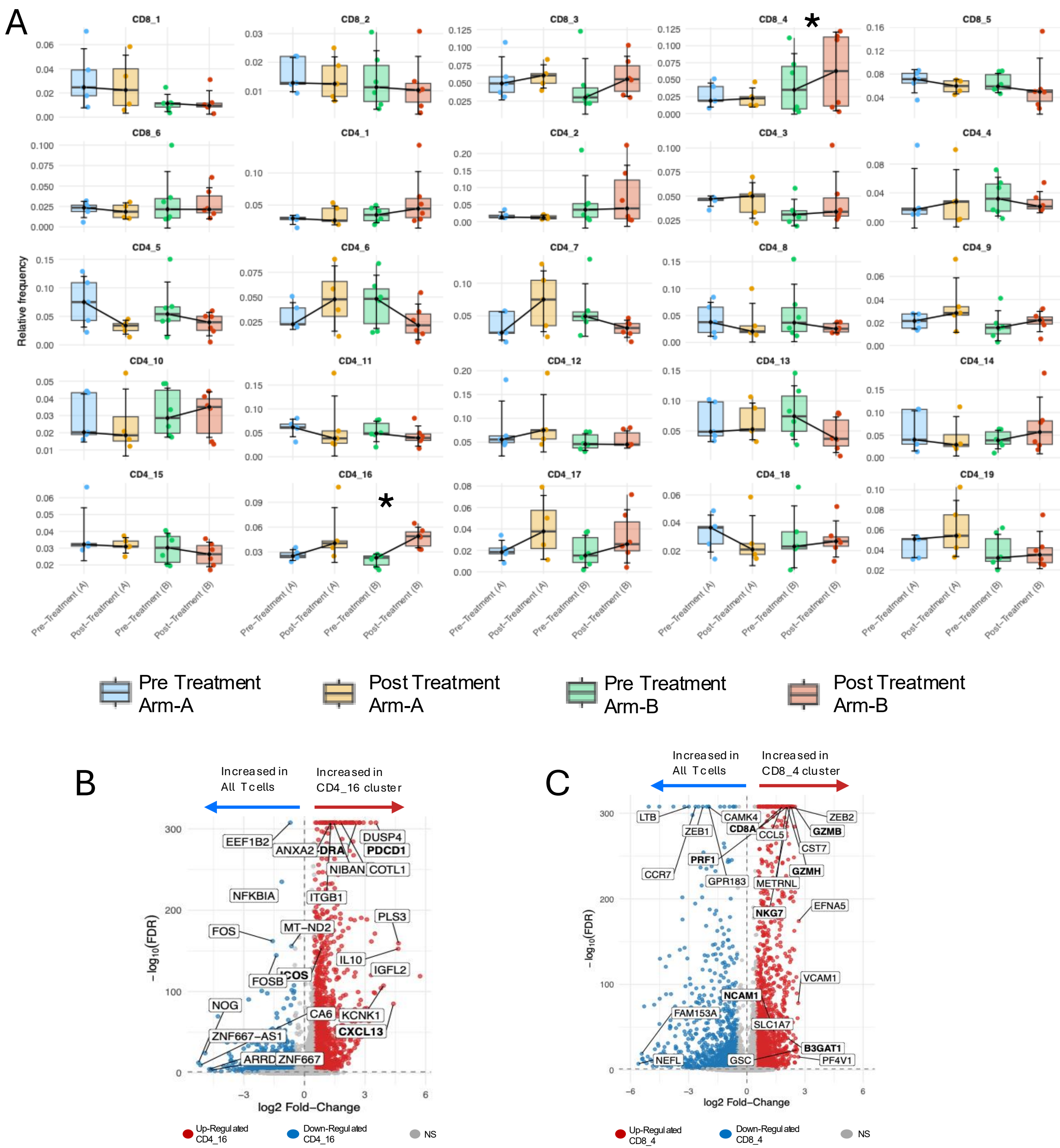

Supplementary Figure 8 | Transcriptional and compositional changes in T cell compartments

(A) Boxplots showing relative frequencies of T cell clusters in Arm-A and Arm-B, comparing pretreatment and post-treatment samples.

(B) Volcano plot depicting differentially expressed genes in the CD4_16 cluster compared with all other T cells.

(C) Volcano plot depicting differentially expressed genes in the CD8_4 cluster compared with all other T cells.

Supplementary Figure 9

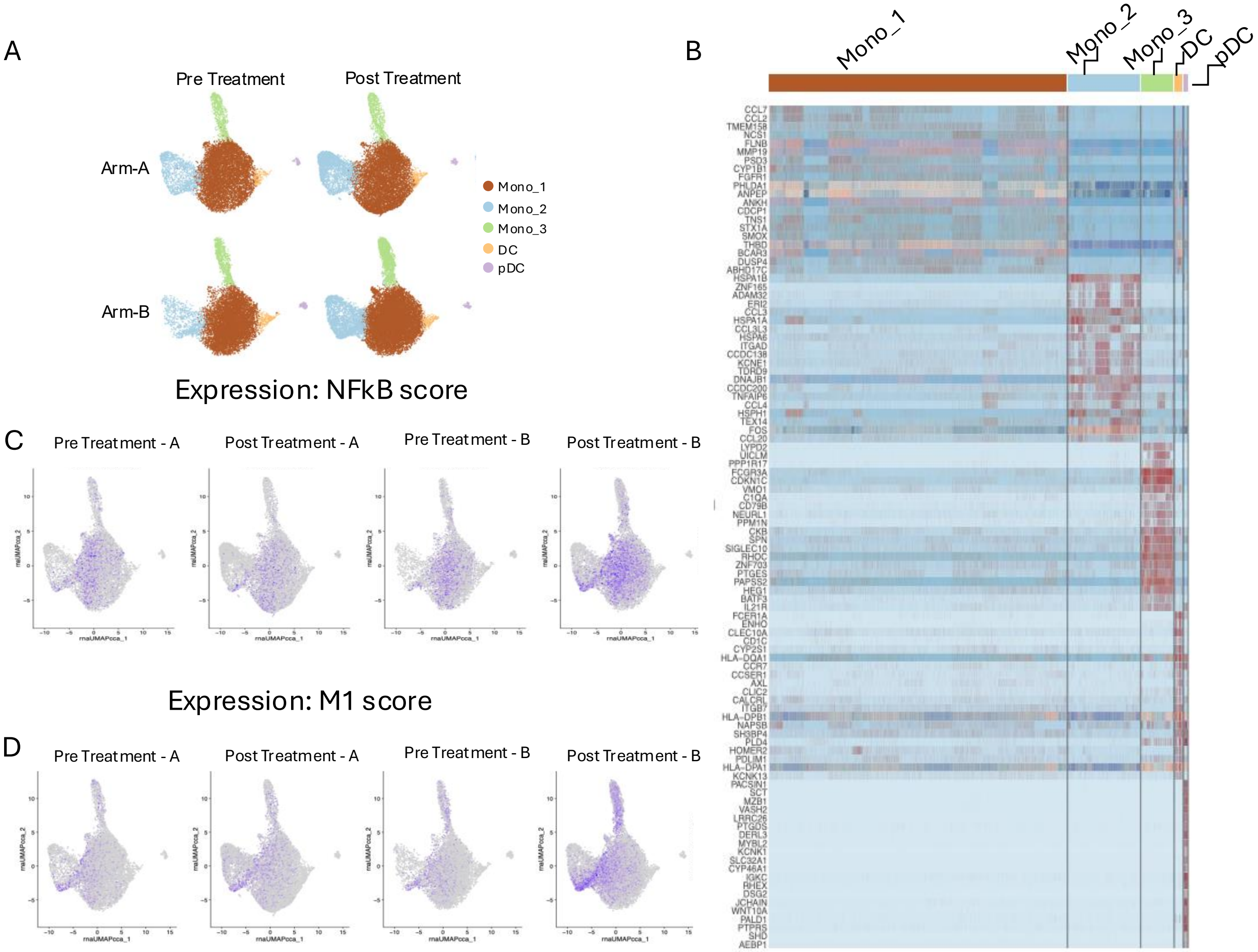

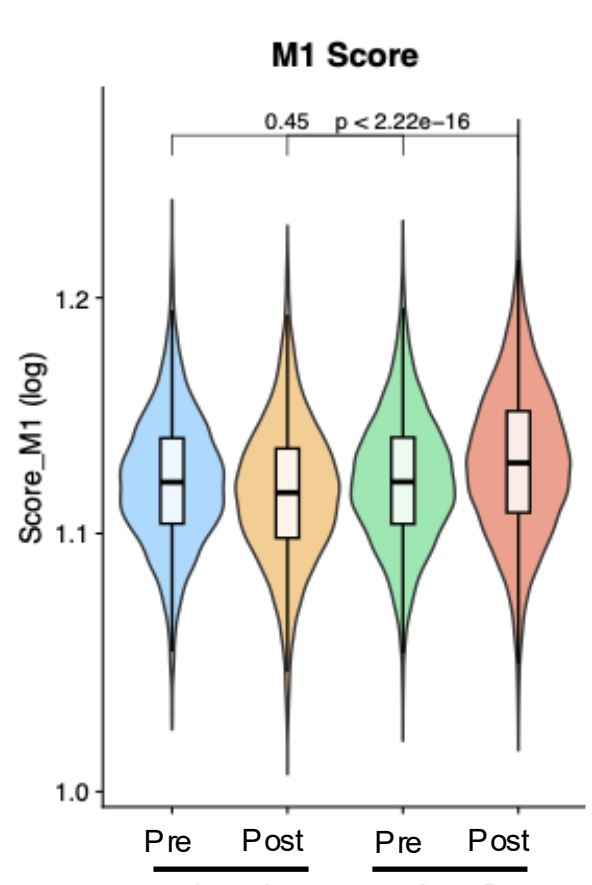

Supplementary Figure 9 | Remodeling of myeloid cell populations following treatment

(A) UMAP visualization of transcriptionally defined myeloid cell clusters.

(B) Heatmap showing differentially expressed genes across myeloid clusters.

(C) Feature plot of myeloid cells highlighting cells within the 80th percentile of the NF-κB signature score.

(D) Feature plot (top) showing myeloid cells within the 80th percentile of the M1 polarization score, with corresponding violin plots (bottom) summarizing M1 scores across myeloid populations.

Supplementary Figure 10

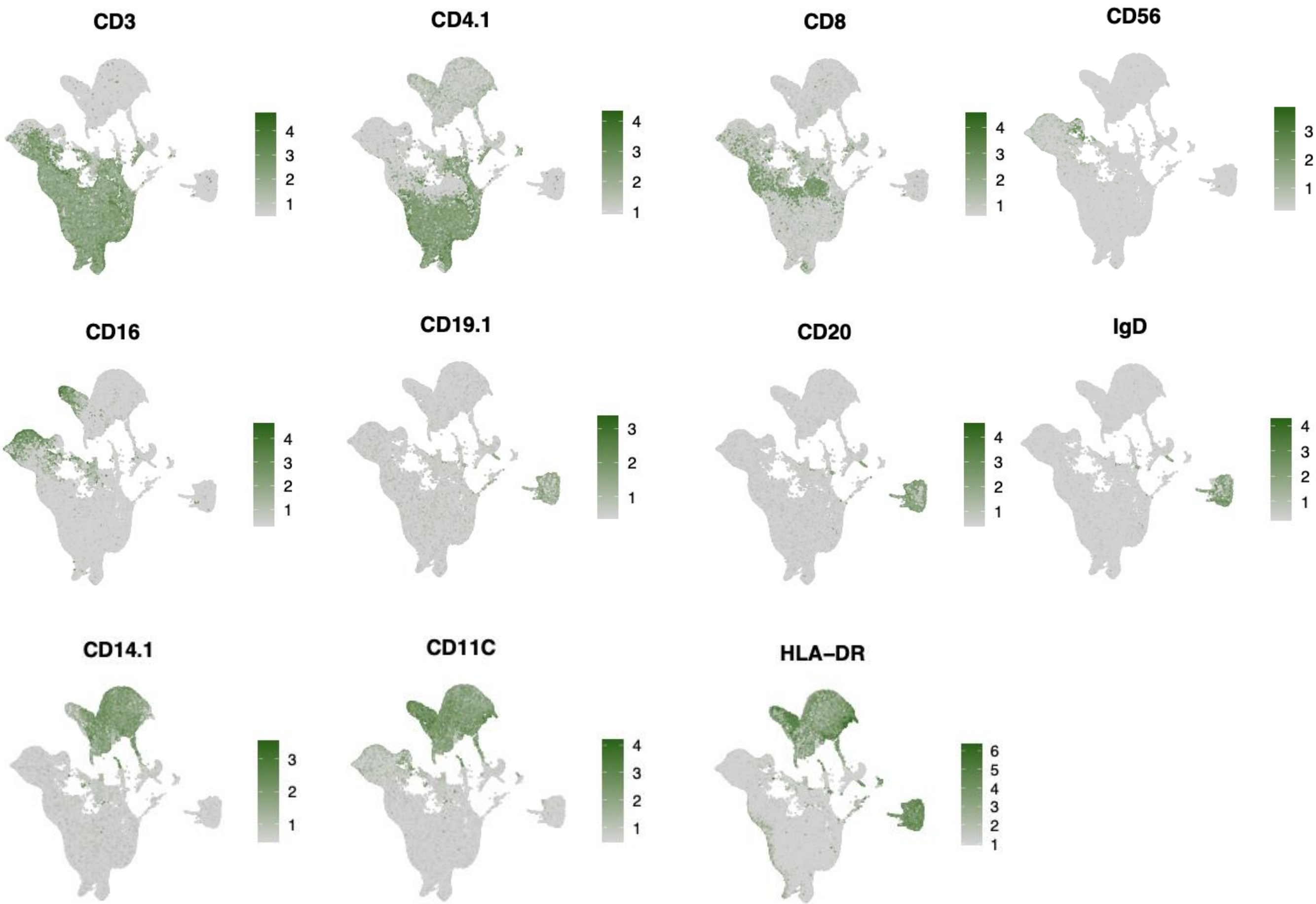

Supplementary Figure 10 | ADT expression profile
Feature plots showing ADT expression profile of main cell-type markers

Supplementary Table 1: Patterns of irAEs

	Pt #	irAE Grade and Involved Organ
**Arm A**		
	002	G3 Colitis
	005	G4 Hepatitis, G2 Thyroid, G1 Colitis, G1 Skin
	007	G2 Pulmonary, G1 Rheumatologic
	013	G3 Colitis, G1 Thyroid
	014	G2 Hepatitis, G2 Rheumatologic
	010	G2 Pulmonary
	004	G1 Thyroid, G1 Adrenal, G1 Skin
**Arm B**		
	008	G3 Hepatitis, G2 Thyroid
	012	G2 Colitis
	015	G2 Colitis
	003	G1 Colitis
	006	G1 Colitis, G1 Thyroid, G1 Rhuematologic
	009	G1 Hepatitis
	001	None

* none of the patients had a prior diagnosis of autoimmunity or chronic steroid use.
# Abdomen includes liver, adrenal, spleen, peritoneal and retroperitoneal. Others include breast and muscle
Onset of hypersensitivity with first dose in 4 patients and $\geq$second dose in 2 additional patients

Supplementary Table 6. Antibodies used for mass cytometry analysis:

Antibody	Clone
CD45	HI30
CD8	RPA-T8
CD11b	ICRF44
CD33	WM53
CCR6	11A9
CD40	5C3
HLADR	L243
CD69	FN50
CD4	RPA-T4
IgD	IA62
CD14	RMO52
CD127	A019D5
CD138	MI15
ICOS	C398.4A
CD21	BL13
CD45RA	HI100
TIGIT	MBSA43
CD27	L128
CXCR3	G025H7
CD11c	Bu15
CCR7	G043H7

Antibody	Clone
CD28	CD23.2
CD45RO	UCHL1
PD-1	EH12.2H7
NKG2D	ON72
CD19	HIB19
CD3	UCHT1
CXCR5	RF8B2
IgM	MHM-88
CD20	2H7
CD25	M-A251
CD38	HIT2
CD56	HCD56
CD16	3G8
GZN	GB11
Tcf1	7F11A10
Tbet	4B10
Foxp3	PCH101
Bcl6	K11291
GATA3	TWAJ
Ki67	B56
